# The Role of Exosomes in Health and Disease

**DOI:** 10.3390/ijms231911011

**Published:** 2022-09-20

**Authors:** Vincenzo Lionetti

**Affiliations:** 1 Unit of Translational Critical Care Medicine, Scuola Superiore Sant’Anna, 56127 Pisa, Italy; v.lionetti@santannapisa.it; 2 UOSVD Anesthesiology and Intensive Care Medicine, Fondazione Toscana “Gabriele Monasterio”, 56124 Pisa, Italy

Who would have thought that the discovery made by researchers at Washington University [1] and McGill University [2] almost 40 years ago would help us to fully understand the adaptive and maladaptive responses of a tissue to a change in the extracellular microenvironment? At the time, it was surprising to observe how maturing blood reticulocytes secreted transferrin receptors within hitherto unexpected tiny membranous vesicles, later named exosomes by Dr. Rose M Johnstone. Today, we know that nanovesicles release is an extraordinary mode of communication of all cells in health and disease throughout life [3,4,5], even in dying cells [6]. However, not all extracellular vesicles (EVs) are the same. There is growing evidence that exosomes (size 30–150 nm), once they are released externally from endosomes, carry varieties of signals produced by the sorting of different proteins, oligonucleotides (e.g., mRNA, microRNA, circRNA, mtDNA), or metabolites (e.g., lipids, amino acids), even in plants. 

Of note, Woith et al. [7] have demonstrated that lipophilic compounds are associated with nanovesicles, while more hydrophilic structures are not consistently found. We can consider this finding of particular interest since plant cells secrete EVs using similar mechanisms as mammal cells. Moreover, plant-derived exosome content could contribute to the potential protective effects of functional foods that enrich our diet as major regulators of the human epigenome [8].

Despite the large number of studies published so far, further investigation of the mechanisms underlying exosomal regulation of organ function is needed to better highlight its clinical relevance, even in critically ill patients [9]. Indeed, we cannot exclude that the efficiency of this type of intercellular communication even depends on the susceptibility of recipient cells toward selective exosomes a under specific microenvironment. For example, some conventional drugs may increase the release of antiapoptotic exosomes targeting cardiomyocytes [10]. Moreover, EV trafficking is involved in inter-tissue communication during exercise, delivering a wide range of adhesion proteins [11]. Beyond this initial evidence, it is still unclear how an external chemical and/or physical stimulus changes the content of an exosome or interferes with its release and internalization under physiological and pathological conditions. We cannot ignore, however, that changes in the circulating levels of exosomes can play a theranostic and prognostic key role in patients with cardiovascular diseases, even in those undergoing cardiac surgery [12,13,14]; acute lung injury/acute respiratory distress syndrome [15]; neurodegenerative disorders [16]; COVID-19 [17]; and other severe diseases, as summarized in this Special Issue. Levin et al. [18] demonstrated that circulating EVs in patients with beta thalassemia major contain high levels of miR-144-3p, a regulator of erythropoiesis, which leads to the proliferation of bone marrow mesenchymal stem cells and reduced survival of endothelial, pancreatic, and hepatic cells, contributing to the onset of organ dysfunction. As a result, there is an increasing need to draft international guidelines to enable exosome-based liquid biopsy in clinical practice and to develop new assays to better quantify and characterize exosomes in both preclinical laboratories and in hospital settings [19]. In light of this topic, Mason et al. [20] have described a unique chip-based approach for the real-time monitoring of nanovesicles crosstalk between different cell populations that are physically separated by an extracellular matrix-mimicking Matrigel under physiologically relevant conditions. Therefore, the use of a multimodal platform to study exosome-mediated intercellular communication has also revealed their potential dose-dependent therapeutic value, which is dependent on the cell source, timing, and mode of administration. For the first time, Barile et al. [21] have shown the dose-dependent inhibition of cardiomyocyte apoptosis by extracellular vesicles derived from cardiac progenitor cells, but not fibroblast-derived exosomes. Thanks to the most recent translational studies, exosomes are considered a new horizon in the cell-free therapy of otherwise incurable diseases, although clinical data on the kinetics and dynamics of a single dose of exosomes are lacking. Finally, exosomes have been proposed as drug transporters for precision medicine, although their use for this purpose has been limited by the lack of efficient drug-loading methods. In this regard, Romano et al. [22] have demonstrated that the manipulation of vesicles curvature by high-pressure homogenization successfully improves and shortens the exosome encapsulation process of the bioactive agent to less than 1 h, preserving their morphological integrity and biological identity. This technology could also be effective in facilitating the development of a new multi-modal molecular imaging approach to guide therapy and surgery by encapsulating the contrast agents or nanoparticles and exploiting the inherent echogenicity of exosomes at the same time [23].

More extensive multidisciplinary studies are needed to fill in the gaps and to overcome the limitations of available technologies in order to reposition exosomes in the first line for their use in the nanobiotheranostic field. 

The purpose of this research topic was to bring together state-of-the-art original research and review articles on the latest evidence in the field of exosome-based applications. It is hoped that in the future, readers of this Special Issue will be inspired to develop more effective and less invasive precision approaches to promote the wide use of exosomes at bedside.
